# Epidemiology of non-steroidal anti-inflammatory drugs consumption in Spain. The MCC-Spain study

**DOI:** 10.1186/s12889-018-6019-z

**Published:** 2018-09-21

**Authors:** Inés Gómez-Acebo, Trinidad Dierssen-Sotos, María de Pedro, Beatriz Pérez-Gómez, Gemma Castaño-Vinyals, Tania Fernández-Villa, Camilo Palazuelos-Calderón, Pilar Amiano, Jaione Etxeberria, Yolanda Benavente, Guillermo Fernández-Tardón, Inmaculada Salcedo-Bellido, Rocío Capelo, Rosana Peiró, Rafael Marcos-Gragera, José M. Huerta, Adonina Tardón, Aurelio Barricarte, Jone-Miren Altzibar, Jessica Alonso-Molero, Verónica Dávila-Batista, Nuria Aragonés, Marina Pollán, Manolis Kogevinas, Javier Llorca

**Affiliations:** 1 CIBER Epidemiología y Salud Pública, Madrid, Spain; 2 Facultad de Medicina, Universidad de Cantabria – IDIVAL, Avda Herrera Oria s/n, 39011 Santander, Spain; 3 Department of Obstetrics and Gynecology, Nuevo Belén University Hospital, Madrid, Spain; 4 Environmental and Cancer Epidemiology Unit, National Centre for Epidemiology, Carlos III Institute of Health (Instituto de Salud Carlos III–ISCIII), Avda. Monforte de Lemos, 5, 28029 Madrid, Spain; 5 Institute of Health Research “Puerta de Hierro”, IDIPHIM, Madrid, Spain; 6 ISGlobal, Centre for Research in Environmental Epidemiology (CREAL), Barcelona, Spain; 7 Universitat Pompeu Fabra (UPF), Barcelona, Spain; 8 IMIM (Hospital del Mar Medical Research Institute), Barcelona, Spain; 9 Institute of Biomedicine (IBIOMED), University of León, León, Spain; 10 Public Health Division of Gipuzkoa, BioDonostia Research Institute, San Sebastian, Spain; 11 Department of Statistics and O.R., INAMAT, Public University of Navarre, Pamplona, Spain; 12 Unit of Infections and Cancer, Cancer Epidemiology Research Programme, IDIBELL, Catalan Institute of Oncology, Barcelona, Spain; 13 University of Oviedo, Oviedo, Spain; 14 Universidad de Granada, Granada, Spain; 15 Centro de Investigación en Recursos Naturales, Salud, y Medio Ambiente. (RENSMA), Universidad de Huelva, Huelva, Spain; 16 Dirección General de Salud Pública, Fundación para el fomento de la investigación sanitaria y biomédica de la Comunidad Valenciana, FISABIO-Salud Pública, Valencia, Spain; 17 Unitat d’Epidemiologia i Registre de Càncer de Girona (UERCG), Pla Director d’Oncologia, Institut Català d’Oncologia, Institut d’Investigaciò Biomèdica de Girona (IdIBGi), Universitat de Girona, Girona, Spain; 18 Department of Epidemiology, Murcia Regional Health Council, IMIB-Arrixaca, Murcia, Spain; 19 Navarra Public Health Institute, Pamplona, Spain; 20 Navarra Institute for Health Research (IdiSNA), Pamplona, Spain; 21 Osakidetza-Basque Health Service, BioDonostia Research Institute, Donostia, Spain; 22 Epidemiology Section, Public Health Division, Department of Health of Madrid, Madrid, Spain

**Keywords:** Aspirin, Cardiovascular risk, Gastrointestinal bleeding, Non-steroidal anti-inflammatory drugs, Propionates

## Abstract

**Background:**

Non-steroidal anti-inflammatory drugs (NSAIDs) are widely used despite their risk of gastrointestinal bleeding or cardiovascular events. We report the profile of people taking NSAIDs in Spain, and we include demographic factors, health-related behaviours and cardiovascular disease history.

**Methods:**

Four thousand sixtyparticipants were selected using a pseudorandom number list from Family Practice lists in 12 Spanish provinces. They completed a face-to-face computerized interview on their NSAID consumption, demographic characteristics, body mass index, alcohol and tobacco consumption and medical history. In addition, participants completed a self-administered food-frequency and alcohol consumption questionnaire. Factors associated with ever and current NSAID consumption were identified by logistic regression.

**Results:**

Women consumed more non-aspirin NSAIDs (38.8% [36.7–41.0]) than men (22.3 [20.5–24.2]), but men consumed more aspirin (11.7% [10.3–13.2]) than women (5.2% [4.3–6.3]). Consumption of non-aspirin NSAIDs decrease with age from 44.2% (39.4–49.1) in younger than 45 to 21.1% (18.3–24.2) in older than 75, but the age-pattern for aspirin usage was the opposite. Aspirin was reported by about 11% patients, as being twice as used in men (11.7%) than in women (5.2%); its consumption increased with age from 1.7% (< 45 years old) to 12.4% (≥75 years old). Aspirin was strongly associated with the presence of cardiovascular risk factors or established cardiovascular disease, reaching odds ratios of 15.2 (7.4–31.2) in women with acute coronary syndrome, 13.3 (6.2–28.3) in women with strokes and 11.1 (7.8–15.9) in men with acute coronary syndrome. Participants with cardiovascular risk factors or diseases consumed as much non-aspirin NSAID as participants without such conditions.

**Conclusions:**

Non-aspirin NSAIDs were more consumed by women and aspirin by men. The age patterns of aspirin and non-aspirin NSAIDs were opposite: the higher the age, the lower the non-aspirin NSAIDs usage and the higher the aspirin consumption. People with cardiovascular risk factors or diseases consumed more aspirin, but they did not decrease their non-aspirin NSAIDs usage.

**Electronic supplementary material:**

The online version of this article (10.1186/s12889-018-6019-z) contains supplementary material, which is available to authorized users.

## Background

Non-steroidal anti-inflammatory drugs (NSAIDs) are one of the most used therapeutic groups of agents; they can be obtained over-the-counter in many countries, and they are used for a wide variety of indications, including short-term and long-term treatment of pain, traumatisms, inflammatory diseases such as arthritis, rheumatoid arthritis and many others. On the other hand, NSAIDs can be responsible for several well-known side effects, comprising upper gastrointestinal bleeding [1] and cardiovascular disease [2]. While gastrointestinal haemorrhage would partially be prevented by adding proton-pump inhibitors to NSAIDs [3, 4], there is still some controversy regarding the differences in cardiovascular risk among the NSAID family [5].

Although the consumption of NSAIDs in Spain has decreased from 43.1 in the year 2013 to 37.9 in 2016, and this decrease in consumption was observed in all subgroups [6]; some studies have shown a trend towards increasing NSAID usage in developed countries [7, 8]. Little is known, however, about the medical characteristics of the consumers. In this way, medical records would be insufficient to establish patient profile, as a relevant NSAID amount is traded over-the-counter. Demographic characteristics are associated with different adverse effect risks. For instance, gastrointestinal haemorrhage is more frequent in elder people taking NSAIDs than in youngsters [1], and similar considerations could are responsible for cardiovascular effects [2]. Moreover, some health-related behaviours, such as alcohol [9] or -we speculate- tobacco usage, if associated with NSAID consumption, could potentiate their risk of cardiovascular episodes or gastrointestinal bleeding. Thus, Chi et al. observed that the proportions of patients with concomitant antiplatelet drugs, H pylori infection and status of smoking were also considerably higher in GI (gastrointestinal) bleeding group compared to non-GI bleeding group GI bleeding group associated with NSAIDs drugs [10] and Sostres et al. also observed that a higher risk of upper GI bleeding was associated with current or past smoking habit and previous history of peptic ulcer [11].

The aim of this study is to describe demographic characteristics related to NSAID consumption in the adult population in Spain, as well as health-related behaviors and cardiovascular risk factors. In order to do this, we analysed the control sample (about 4000 subjects) in the MCC-Spain project, a multi-centre case-control study carried out in Spain.

## Methods

MCC-Spain is a case-control study on cancer carried out in 12 Spanish provinces: Asturias, Barcelona, Cantabria, Girona, Granada, Gipuzkoa, Huelva, León, Madrid, Murcia, Navarra, and Valencia [12]. More than 10,000 patients were recruited from 2009 to 2012, including cases of colorectal, breast, prostate or gastric cancer, and chronic lymphoid leukaemia, and 4062 controls frequency matched by age, sex and area of recruitment. In this article, only the control sample will be analysed, so all references to patients, subjects or participants from here on refer to the control sample. The study design, sample size and data gathering were planned for the case-control study.

Participants were recruited using computer-generated pseudorandom numbers from the list of patients assigned to general practice clinics. Selected people were contacted by phone; if contact with the selected person was not possible after a minimum of five tries at different times of the day, or if he/she refused to participate, the following person on the list was approached. In the Spanish Health System, every inhabitant is assigned to a general practice clinic irrespective of whether he or she does attend to that clinic; therefore, selecting by random from those lists did not introduce a bias towards sick people. Participants who agreed to partake in the study signed an informed consent before the face-to-face interview, and the protocol of MCC-Spain was approved by the local Ethics Committees of participating institutions (Comité Ético de Investigación Clínica (CEIC) del Instituto Municipal de Asistencia Sanitaria de Barcelona; CEIC del Hospital Universitario de Bellvitge; CEIC de Navarra; CEIC del Hospital Universitario La Paz; CEIC del Hospital Universitario Ramón y Cajal; CEIC de Cantabria; CEIC de Gipuzkoa; CEIC de Girona; Comité de Ética de la Investigación de la Provincia de Huelva; CEIC de León; Comité Ético de Investigación del Principado de Asturias), in conformity to the principles of the Declaration of Helsinki. The database was registered in the Spanish Agency for Data Protection (no. 2102672171).

A structured computerized epidemiological questionnaire was administered by trained personnel in a face-to-face interview to get information on demographics, anthropometrics, family history of cancer, history of diseases, drug consumption, occupational history, health behaviours, and reproductive factors [13]. Usage of NSAIDs was specifically asked about using a detailed questionnaire including the specific NSAID, age at beginning, age when end duration of consumption and current consumption; a participant was considered as having taken a specific NSAID if she/he reported to have taken at least 30 doses. We carried out separated analyses for ever and current consumers of NSAIDs; current consumption could be consequence of recent conditions, while ever consumption better represents cumulative exposure to NSAIDs but it could be more prone to recall bias.

On the other hand, participants were provided with a semi-quantitative Food Frequency Questionnaire (FFQ) previously validated in the Spanish population [14], which included questions on alcohol consumption both currently and at 30–40 years old [15]. The FFQ was self-administered and returned by mail or filled out face to face within a period not exceeding 15 days after the interview [13]. Only 3509 participants answered this questionnaire. Alcohol consumption was asked for every type of beverage; for instance, we asked “How often do you drink one glass of red wine?”, giving the options: never or less than 1 time per month / 1–3 per month / 1–2 per week / 3–4 per week / 5–6 per week / 1 per day / 2–3 per day / 2–3 per day / 4 or more per day. Then we assumed a glass of wine being 100 cL, containing 12% alcohol. Average alcohol drinking was classified in abstainer (less than one drink per month), category I (0–19.9 g /day for women and 0–39.9 g/day for men), category II (20–39.9 g/day for women, 40–59.9 g/day for men) and category III (≥40 g/day for women, ≥60 g/day for men), according to the comparative risk assessment module of the Global Burden of Disease [16]. For instance, it would be necessary to take 4 glasses of wine or 2 cups of whisky to reach 40 g of alcohol. NSAIDs were classified according to the Anatomical and Therapeutic Classification of Drugs (ATC) in aspirin (ATC code N02BA01), butilpirazone (M01aa), acetic derivatives (M01ab), oxicams (M01 ac), propionates (M01ae), coxibs (M01ah) and others (M01ax).

A separated analysis was carried out to ascertain NSAID consumption in people with cardiovascular diseases or risk factors, as current clinical guidelines point out the increase of cardiovascular risk associated with non-aspirin NSAIDs.

Proportions and their 95% confidence intervals (CI) were estimated assuming a binomial distribution. Variables associated with NSAID consumption were identified by binomial logistic regression; its results are displayed as odds ratios (OR) with 95% CI. The statistical package Stata 14/SE was used for the analysis (Stata Corp, College Station, Tx, US).

## Results

Characteristics of the 4060 controls included in this analysis are reported in Table 1. They were 2023 women and 2037 men, with ages ranging 22–85; 49.4% had reached secondary or university education. 61.8% subjects were overweight or obese, 19.2% were current smokers and, when being 30–40 years old, 16% had an average alcohol consumption higher than 20 g/day in women and 40 g/day in men. About one participant in four suffered arthritis, 10% had chronic cephalalgia and 5.6%, gout. Arthritis and chronic cephalalgia were more frequent in women.

**Table 1:** Descriptive analysis of sociodemographic variables

Variable	Category	Total	Women (*N* = 2023, 49.8%)	Men (*N* = 2037, 50.2%)
		Frequency (%) or MEAN ± SD	Frequency (%) or MEAN ± SD	Frequency (%) or MEAN ± SD
Age (years)	Continuous	62.9 ± 12.1	59.3 ± 13.3	66.5 ± 9.6
Age (years)	22–44	421 (10.4)	357 (17.7)	64 (3.1)
	45–54	579 (14.3)	428 (21.2)	151 (7.4)
	55–64	1013 (25.0)	444 (22.0)	569 (27.9)
	65–74	1278 (31.5)	476 (23.5)	803 (39.4)
	75–85	769 (18.9)	318 (15.7)	452 (22.2)
Education level	Lower than primary	755 (18.6)	372 (18.4)	383 (18.8)
	Primary education	1301 (32.0)	616 (30.5)	686 (33.6)
	Secondary education	1172 (28.9)	616 (30.5)	556 (27.3)
	University	832 (20.5)	419 (20.7)	414 (20.3)
Body mass index (kg/m^2^)	< 18.5	54 (1.3)	45 (2.29	9 (0.4)
	18.5–24.9	1498 (36.9)	946 (46.8)	553 (27.1)
	25–29.9 (overweight)	1661 (40.9)	640 (31.6)	1022 (50.1)
	≥30 (obesity)	847 (20.9)	392 (19.4)	455 (22.3)
Smoking	No smoker	1812 (44.6)	1236 (61.1)	576 (28.3)
	Current smoker	780 (19.2)	375 (18.5)	405 (19.9)
	Former smoker	1468 (36.2)	1057 (51.8)	411 (20.4)
Alcohol consumption at recruitment (g/day)	Abstainers	939 (26.8)	637 (37.0)	302 (16.9)
	0–19.9 (women), 0–39.9 (men)	2295 (65.4)	999 (58.1)	1296 (72.4)
	20–39.9 (women), 40–59.9 (men)	203 (5.8)	74 (4.3)	129 (7.2)
	≥40 (women), ≥60 (men)	72 (2.0)	9 (0.5)	63 (3.5)
Alcohol consumption when 30–40 years old (g/day)	0	903 (25.7)	657 (38.3)	246 (13.7)
	0–19.9 (women), 0–39.9 (men)	2047 (58.3)	951 (55.3)	1096 (61.2)
	20–39.9 (women), 40–59.9 (men)	300 (8.6)	86 (5.0)	214 (12.0)
	≥40 (women), ≥60 (men)	259 (7.4)	24 (1.4)	235 (13.1)
Number of births(women)	0		375 (18.5)	
	1–2		1085 (53.6)	
	> 2		563 (27.9)	
Menopausal status (women)	Premenopausal		650 (32.1)	
	Postmenopausal		1373 (67.9)	
Chronic disease involving pain	Arthritis	1005 (24.9)	626 (31.1)	379 (18.7)
	Gout	225 (5.6)	24 (1.2)	201 (9.9)
	Chronic cephalalgia	414 (10.2)	307 (15.3)	107 (5.3)
	Arthritis, gout or chronic cephalalgia	1425 (35.1)	816 (40.3)	609 (29.9)
Cardiovascular disease or risk factor	Diabetes mellitus	594 (14.7)	191 (9.5)	403 (19.8)
	Hypertension	1513 (37.4)	587 (29.2)	926 (45.6)
	Hypercholesterolemia	1344 (33.3)	580 (28.9)	764 (37.7)
	Acute coronary syndrome	312 (7.7)	58 (2.9)	254 (12.5)
	Stroke	151 (3.7)	53 (2.6)	94 (4.8)
	Other circulatory diseases	628 (15.5)	330 (16.4)	298 (14.7)

**Fig. 1:**
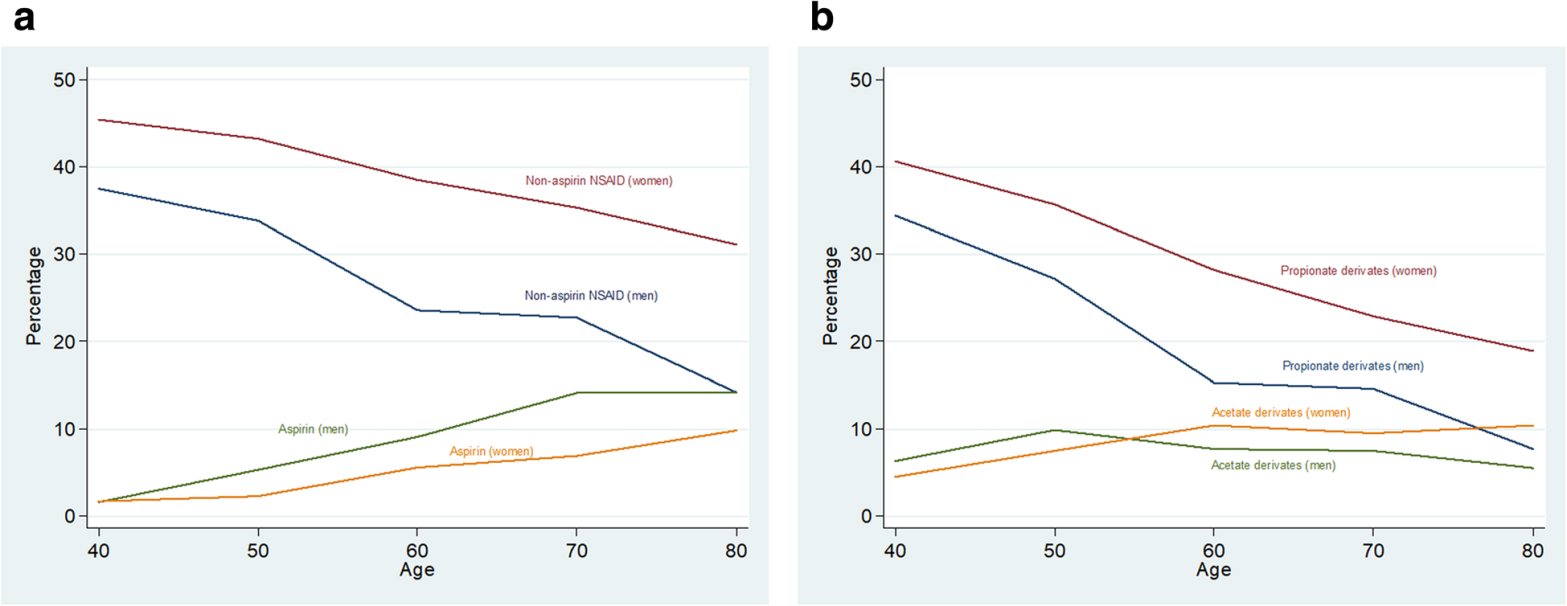
Anytime consumption of NSAID by age and sex. 1**a**: Aspirin and non-aspirin NSAID. 1**b**: Propionates and acetate derivates

**Fig. 2:**
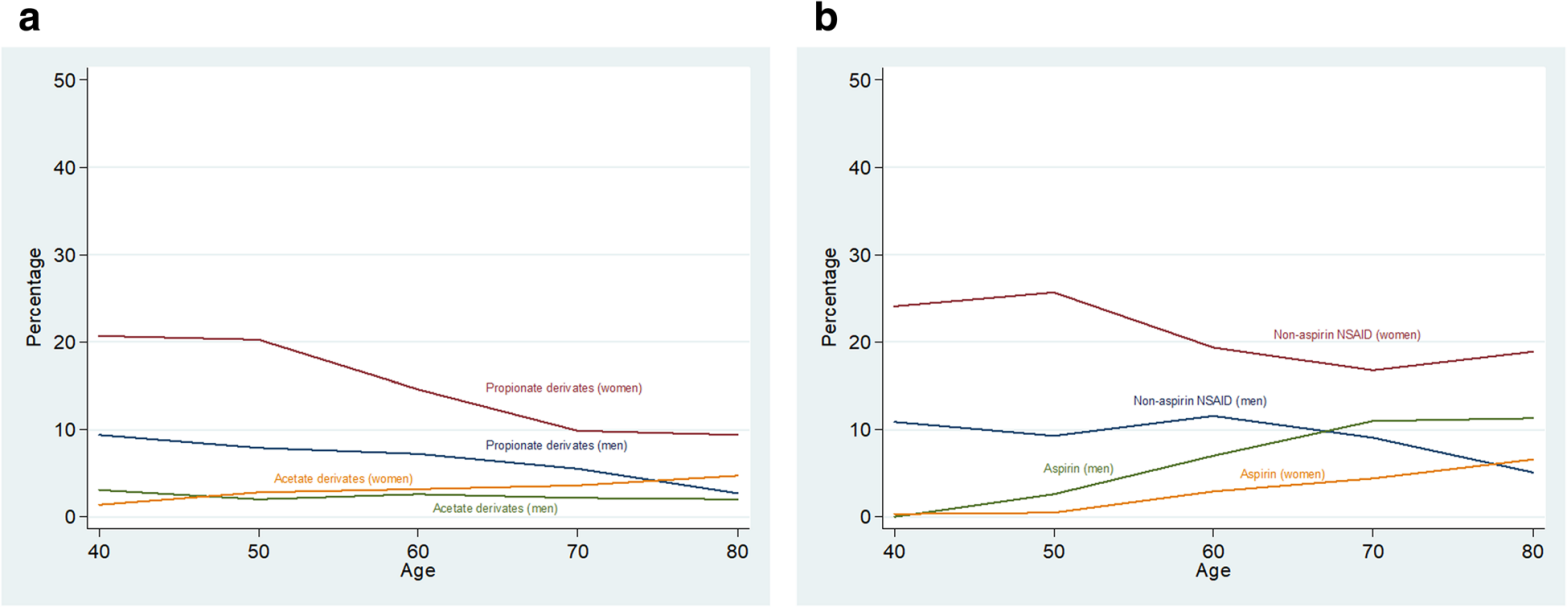
Current consumption of NSAID by age and sex. 1**a**: Aspirin and non-aspirin NSAID. 1**b**: Propionates and acetate derivates

Figure 1 and Additional file 1 reported the ever NSAID consumption frequency by age and sex. About 30% subjects reported non-aspirin NSAID consumption, showing a step-down trend with age, from 44.2% in subjects under 45 to 21.1% in patients over 75. Women consumed NSAID at higher rates than men (38.8% vs. 22.3%); this gender pattern was consistent among all ages. The most consumed NSAID group was propionates (M01ae) (29.2%) with, again, a consistent age and sex pattern: higher consumption in women and in youngsters. Aspirin was reported by about 11% patients, being twice as used in men (11.7%) than in women (5.2%); its consumption increased with age from 1.7% (< 45 years old) to 12.4% (≥75 years old). Acetate derivatives (M01ab) -the third most consumed group- was reported in similar percentages by both sexes, without a neat trend with age. Consumption of the remaining groups was scarce (butylpyrazolidines (M01aa): 0.03%, oxicam (M01 ac): 0.6%, coxib (M01ah): 0.6%, others (M01ax): 2.19%) and we did not carry out additional analyses on them. Consumption of NSAIDs at the time of recruitment is reported in Fig. 2 and Additional file 2. Women used non-aspirin NSAIDs twice as much as men (20.7% for women vs. 9.0% for men); again, this pattern was consistent across age groups, with non-aspirin NSAID consumption decreasing from 22.1% in younger than 45 years to 10.4% in older than 75. Aspirin, however, was more consumed by men (9.0%) than for women (2.9%) and its rates increased with age in both men and women. About two thirds of the non-aspirin NSAID consumption was due to propionates; in men, the consumption declined from 9.4% in younger than 45 years old to 2.7% in men older than 75; in women, the decrease was from 20.7% (< 45 years) to 9.4% (> 75 years).

Consumption by province (Additional file 3) was heterogeneous ranging from 13.4% (in Granada) to 45.1% in Girona) for non-aspirin NSAIDs.

The most frequently ever used specific NSAIDs were ibuprofen (20.4%), aspirin (11%) and diclofenac (6.4%). Only three other drugs were used by more than 1% of people: aciclofenac (1.5%), naproxen (1.4%) and chondroitin sulphate (1.3%). Regarding current consumption, only ibuprofen (9.2%), aspirin (5.9%), diclofenac (2.0%) and chondroitin sulphate (1.0%) reached the 1% cut off (Additional file 4).

### Factors related to NSAIDs ever consumption

Tables 2, 3, 4 and 5 report the factors associated with consumption of the main NSAID groups in men and women, according to the multivariate logistic regression analysis. Non-aspirin NSAID were less consumed in men as age increased (reaching OR = 0.26 in men older than 75 compared to men between 45 and 54 years old) and as education level goes up, being about twice as frequent in men with university level than in men without primary studies; no association was found among non-aspirin NSAID consumption in men and body mass index (BMI)or ethanol consumption. However, men who currently use NSAIDs that are not aspirin were half less likely (OR = 0.49) to be current smokers than non-smokers. If we consider chronic disease involving pain, men who had arthritis multiplied by almost 3 the probability of taking non-aspirin NSAID and doubled its use in other chronic disease involving pain that were not arthritis gout or chronic cephalalgia (Table 2). Similar patterns were found in women, although both age and education levels showed less apparent trends. Number of births and menopausal status were not associated with non-aspirin NSAIDs consumption. Patients with chronic conditions involving pain (arthritis, gout or chronic cephalalgia) consumed non-aspirin NSAIDs four times as much as participants without such conditions. In contrast than men, women who once used NSAIDs without aspirin were approximately 38% more likely to be current smokers than nonsmokers and use in chronic cephalalgia (OR 2.54 for ever or OR 1.66 for current use).

**Table 2:** Factors associated with non-aspirin NSAID consumption: odds ratios and 95% confidence intervals adjusted for the remaining factors in the table and province of recruitment

Variable	Category	Ever consumption		Current consumption	
		Women	Men	Women	Men
Age	< 45	1.50 (1.04–2.15)	1.23 (0.62–2.44)	1.17 (0.78–1.75)	1.57 (0.56–4.42)
	45–54	1 (ref)	1 (ref)	1 (ref)	1 (ref)
	55–64	0.58 (0.40–0.85)	0.53 (0.34–0.83)	0.62 (0.41–0.96)	1.27 (0.63–2.55)
	65–74	0.39 (0.26–0.59)	0.45 (0.29–0.70)	0.39 (0.24–0.62)	0.76 (0.38–1.54)
	≥75	0.39 (0.24–0.62)	0.26 (0.16–0.43)	0.46 (0.27 (0.78)	0.37 (0.17–0.84)
BMI	< 18.5	1.06 (0.53–2.15)	1.75 (0.23–13.6)	0.39 (0.14–1.07)	3.50 (0.28–44.3)
	18.5–24.9	1 (ref)	1 (ref)	1 (ref)	1 (ref)
	25–29.9	0.94 (0.73–1.21)	0.99 (0.74–1.32)	0.94 (0.70–1.27)	0.90 (0.60–1.37)
	≥30	1.38 (1.02–1.88)	0.85 (0.59–1.22)	1.30 (0.92–1.84)	1.06 (0.65–1.74)
Education	Less than primary	1 (ref)	1 (ref)	1 (ref)	1 (ref)
	Primary education	1.31 (0.92–1.85)	1.56 (1.04–2.33)	0.91 (0.61–1.36)	1.11 (0.63–1.98)
	Secondary education	1.21 (0.83–1.76)	2.10 (1.39–3.16)	0.90 (0.58–1.39)	1.30 (0.72–2.33)
	University	1.70 (1.12–2.58)	2.55 (1.65–3.95)	1.29 (0.80–2.07)	2.12 (1.15–3.89)
No. of births (by each birth)		1.04 (0.97–1.12)		1.05 (0.96–1.14)	
Premenopause (ref.: postmenopause)		0.86 (0.61–1.22)		1.15 (0.77–1.70)	
Smoking	No smoker	1 (ref)	1 (ref)	1 (ref)	1 (ref)
	Former smoker	1.20 (0.90–1.59)	1.03 (0.77–1.36)	1.00 (0.72–1.39)	0.98 (0.66–1.45)
	Current smoker	1.38 (1.02–1.88)	0.76 (0.52–1.09)	1.07 (0.76–1.53)	0.49 (0.27–0.88)
Alcohol consumption at recruitment (g/day)					
Abstainers		1 (ref)	1 (ref)	1 (ref)	1 (ref)
0–39.9 (men), 0–19.9 (women)		1.03 (0.76–1.39)	1.26 (0.85–1.88)	0.78 (0.55–1.11)	1.06 (0.58–1.92)
40–59.9 (men), 20–39.9 (women		1.11 (0.57–2.14)	1.60 (0.89–2.89)	0.58 (0.25–1.30)	1.16 (0.49–2.79)
≥ 60 g/day (men), ≥40 g/day (women)		1.22 (0.26–5.68)	1.95 (0.94–4.02)	3.03 (0.56–16.6)	2.69 (0.97–7.44)
Alcohol when 30–40 years old					
Abstainers		1 (ref)	1 (ref)	1 (ref)	1 (ref)
0–39.9 (men), 0–19.9 (women)		1.01 (0.75–1.36)	0.94 (0.62–1.42)	1.30 (0.92–1.84)	1.88 (0.96–3.68)
40–59.9 (men), 20–39.9 (women		1.28 (0.69–2.36)	1.08 (0.64–1.82)	1.93 (0.95–3.93)	2.11 (0.94–4.72)
≥ 60 g/day (men), ≥40 g/day (women)		0.97 (0.37–2.58)	0.81 (0.48–1.36)	1.42 (0.45–4.47)	0.78 (0.32–1.87)
Chronic disease involving pain					
Yes (ref: No)		4.31 (3.37–5.50)	3.60 (2.78–4.67)	3.77 (2.85–4.99)	4.59 (3.18–6.61)

**Table 3:** Factors associated with propionate derivate consumption: odds ratios and 95% confidence intervals adjusted for the remaining factors in the table and province of recruitment

Variable	Category	Ever consumption		Current consumption	
		Women	Men	Women	Men
Age	< 45	1.47 (1.02–2.12)	1.48 (0.73–3.00)	1.17 (0.76–1.79)	1.33 (0.45–3.94)
	45–54	1 (ref)	1 (ref)	1 (ref)	1 (ref)
	55–64	0.54 (0.36–0.80)	0.45 (0.28–0.73)	0.72 (0.45–1.16)	0.85 (0.41–1.76)
	65–74	0.32 (0.21–0.49)	0.40 (0.25–0.65)	0.36 (0.21–0.62)	0.57 (0.27–1.19)
	≥75	0.30 (0.18–0.51)	0.20 (0.11–0.35)	0.38 (0.20–0.71)	0.19 (0.07–0.50)
BMI	< 18.5	1.46 (0.72–2.98)	0.98 (0.10–10.1)	0.33 (0.09–1.13)	–
	18.5–24.9	1 (ref)	1 (ref)	1 (ref)	1 (ref)
	25–29.9	0.81 (0.61–1.07)	0.98 (0.71–1.36)	0.94 (0.67–1.31)	0.98 (0.60–1.62)
	≥30	1.38 (1.00–1.90)	0.81 (0.54–1.23)	1.35 (0.92–1.98)	1.33 (0.75–2.39)
Education	Less than primary	1 (ref)	1 (ref)	1 (ref)	1 (ref)
	Primary education	1.30 (0.89–1.90)	1.83 (1.13–2.96)	0.91 (0.57–1.45)	1.27 (0.63–2.56)
	Secondary education	1.15 (0.76–1.74)	2.17 (1.33–3.55)	0.82 (0.49–1.35)	1.27 (0.63–2.56)
	University	1.42 (0.90–2.23)	2.57 (1.53–4.33)	1.00 (0.58–1.72)	2.00 (0.95–4.24)
No. of births	(by each birth)	1.08 (0.99–1.17)		1.01 (0.91–1.12)	–
Premenopause (ref.: postmenopause)		1.16 (0.80–1.67)		1.57 (1.02–2.43)	–
Smoking	No smoker	1 (ref)	1 (ref)	1 (ref)	1 (ref)
	Former smoker	1.09 (0.81–1.48)	1.05 (0.76–1.46)	1.01 (0.70–1.46)	0.95 (0.60–1.51)
	Current smoker	1.13 (0.82–1.56)	0.74 (0.48–1.14)	1.10 (0.75–1.62)	0.55 (0.28–1.08)
Alcohol consumption at recruitment (g/day)					
Abstainers		1 (ref)	1 (ref)	1 (ref)	1 (ref)
0–39.9 (men), 0–19.9 (women)		1.22 (0.89–1.68)	1.06 (0.67–1.66)	0.75 (0.51–1.10)	0.91 (0.47–1.77)
40–59.9 (men), 20–39.9 (women)		1.41 (0.69–2.87)	1.10 (0.55–2.19)	0.58 (0.22–1.52)	0.98 (0.35–4.48)
≥ 60 g/day (men), ≥40 g/day (women)		0.50 (0.05–4.80)	1.48 (0.64–3.39)	0.98 (0.09–10.9)	1.25 (0.35–4.48)
Alcohol when 30–40 years old					
Abstainers		1 (ref)	1 (ref)	1 (ref)	1 (ref)
0–39.9 (men), 0–19.9 (women)		0.86 (0.63–1.18)	1.22 (0.76–1.96)	1.19 (0.81–1.75)	1.10 (0.54–2.23)
40–59.9 (men), 20–39.9 (women)		0.79 (0.40–1.54)	1.15 (0.63–2.12)	1.29 (0.55–3.01)	1.26 (0.52–3.06)
≥ 60 g/day (men), ≥40 g/day (women)		0.55 (0.17–1.75)	0.94 (0.51–1.73)	0.60 (0.12–3.11)	0.62 (0.24–1.63)
Chronic disease involving pain					
Yes (ref: No)		3.08 (2.37–3.99)	2.22 (1.65–2.98)	2.58 (1.89–3.52)	2.48 (1.61–3.82)

**Table 4:** Factors associated with aspirin consumption: odds ratios and 95% confidence intervals adjusted for the remaining factors in the table and province of recruitment

Variable	Category	Ever consumption		Current consumption	
		Women	Men	Women	Men
Age	< 45	0.62 (0.20–1.96)	0.25 (0.03–2.06)	0.76 (0.06–9.39)	–
	45–54	1 (ref)	1 (ref)	1 (ref)	1 (ref)
	55–64	2.03 (0.81–5.13)	1.54 (0.70–3.40)	2.99 (0.57–15.7)	2.35 (0.81–6.84)
	65–74	2.26 (0.85–6.01)	2.41 (1.12–5.19)	4.90 (0.91–26.3)	3.99 (1.41–11.3)
	≥75	3.59 (1.26–10.2)	3.09 (1.39–6.87)	6.83 (1.21–38.7)	4.74 (1.62–13.9)
BMI	< 18.5	1.77 (0.37–8.38)	–	2.01 (0.24–17.1)	–
	18.5–24.9	1 (ref)	1 (ref)	1 (ref)	1 (ref)
	25–29.9	0.75 (0.41–1.36)	1.02 (0.70–1.47)	0.68 (0.31–1.53)	2.35 (0.81–6.84)
	≥30	1.73 (0.98–3.07)	1.32 (0.85–2.04)	1.42 (0.66–3.05)	1.45 (0.89–2.38)
Education	Less than primary	1 (ref)	1 (ref)	1 (ref)	1 (ref)
	Primary education	0.77 (0.41–1.45)	1.43 (0.90–2.27)	0.62 (0.29–1.30)	1.23 (0.75–2.02)
	Secondary education	0.84 (0.42–1.71)	1.60 (0.98–2.61)	0.34 (0.12–0.94)	1.35 (0.79–2.29)
	University	0.87 (0.38–2.05)	1.56 (0.92–2.65)	0.16 (0.03–0.80)	1.22 (0.68–2.19)
No. of births	(by each birth)	1.25 (1.09–1.42)		1.15 (0.97–1.36)	–
Premenopause	(ref.: postmenopause)	1.21 (0.50–2.93)		0.72 (0.19–2.75)	–
Smoking	No smoker	1 (ref)	1 (ref)	1 (ref)	1 (ref)
	Former smoker	1.33 (0.69–2.58)	1.67 (1.14–2.44)	1.00 (0.36–2.80)	1.80 (1.16–2.79)
	Current smoker	1.89 (0.96–3.71)	1.64 (1.01–2.64)	1.71 (0.63–4.65)	1.85 (1.07–3.19)
Alcohol consumption at recruitment (g/day)					
Abstainers		1 (ref)	1 (ref)	1 (ref)	1 (ref)
0–39.9 (men), 0–19.9 (women)		1.14 (0.62–2.08)	0.81 (0.52–1.27)	1.19 (0.54–2.65)	0.96 (0.58–1.60)
40–59.9 (men), 20–39.9 (women)		0.64 (0.15–2.69)	0.49 (0.22–1.09)	1.05 (0.16–6.98)	0.52 (0.21–1.33)
≥ 60 g/day (men), ≥40 g/day (women)		NA	1.01 (0.41–2.46)	–	1.38 (0.52–3.65)
Alcohol when 30–40 years old					
Abstainers		1 (ref)	1 (ref)	1 (ref)	1 (ref)
0–39.9 (men), 0–19.9 (women)		1.00 (0.55–1.83)	1.73 (1.00–3.00)	1.08 (0.49–2.40)	1.40 (0.77–2.53)
40–59.9 (men), 20–39.9 (women)		2.16 (0.75–6.26)	1.44 (0.72–2.89)	2.61 (0.62–10.9)	1.13 (0.52–2.45)
≥ 60 g/day (men), ≥40 g/day (women)		NA	1.44 (0.74–2.78)	–	1.23 (0.60–2.53)
Chronic disease involving pain					
Yes (ref: No)		1.27 (0.78–2.08)	1.23 (0.89–1.70)	0.71 (0.36–1.36)	1.10 (0.76–1.59)

**Table 5:** Factors associated with acetate acid derivate consumption: odds ratios and 95% confidence intervals adjusted for the remaining factors in the table and province of recruitment

Variable	Category	Ever consumption		Current consumption	
		Women	Men	Women	Men
Age	< 45	1.12 (0.51–2.43)	0.76 (0.22–2.59)	0.86 (0.26–2.81)	2.88 (0.39–21.2)
	45–54	1.00 (ref)	1 (ref)	1 (ref)	1 (ref)
	55–64	0.86 (0.47–1.55)	0.58 (0.28–1.19)	0.73 (0.29–1.84)	1.44 (0.33–6.27)
	65–74	0.66 (0.34–1.26)	0.52 (0.26–1.06)	0.52 (0.18–1.45)	0.96 (0.22–4.19)
	≥75	0.81 (0.39–1.68)	0.48 (0.22–1.06)	0.73 (0.24–2.24)	1.20 (0.25–5.62)
BMI	< 18.5	0.27 (0.03–2.13)	2.62 (0.17–40.8)	0.78 (0.09–6.66)	–
	18.5–24.9	1.00 (ref)	1 (ref)	1 (ref)	1 (ref)
	25–29.9	1.30 (0.84–2.00)	1.22 (0.76–1.97)	1.67 (0.87–3.22)	0.49 (0.22–1.06)
	≥30	1.57 (0.97–2.56)	1.32 (0.74–2.34)	1.27 (0.57–2.83)	0.81 (0.34–1.94)
Education	Less than primary	1 (ref)	1 (ref)	1 (ref)	1 (ref)
	Primary education	1.14 (0.67–1.96)	1.34 (0.70–2.57)	0.69 (0.30–1.58)	1.45 (0.46–4.57)
	Secondary education	0.86 (0.46–1.59)	2.27 (1.20–4.28)	0.58 (0.23–1.50)	1.93 (0.63–5.89)
	University	1.41 (0.73–2.71)	2.15 (1.09–4.27)	1.29 (0.49–3.35)	2.40 (0.73–7.87)
No. of births	(by each birth)	0.97 (0.85–1.10)		1.12 (0.94–1.33)	–
Premenopause (ref.: postmenopause)		0.45 (0.23–0.86)		0.58 (0.22–1.49)	–
Smoking	No smoker	1 (ref)	1 (ref)	1 (ref)	1 (ref)
	Former smoker	1.20 (0.73–1.99)	0.91 (0.57–1.44)	0.94 (0.42–2.08)	1.66 (0.71–3.84)
	Current smoker	1.72 (1.03–1.99)	0.91 (0.51–1.63)	1.33 (0.59–2.99)	0.96 (0.31–3.02)
Alcohol consumption at recruitment (g/day)					
Abstainers		1 (ref)	1 (ref)	1 (ref)	1 (ref)
0–39.9 (men), 0–19.9 (women)		0.90 (0.55–1.48)	1.14 (0.59–2.19)	1.01 (0.46–2.21)	0.88 (0.27–2.81)
40–59.9 (men), 20–39.9 (women)		1.27 (0.50–3.24)	1.98 (0.83–4.73)	0.85 (0.19–3.81)	1.28 (0.27–6.08)
≥ 60 g/day (men), ≥40 g/day (women)		1.80 (0.26–12.3)	1.40 (0.46–4.31)	3.43 (0.18–64.1)	2.80 (0.50–15.6)
Alcohol when 30–40 years old					
Abstainers		1 (ref)	1 (ref)	1 (ref)	1 (ref)
0–39.9 (men), 0–19.9 (women)		1.24 (0.75–2.04)	0.75 (0.39–1.41)	1.35 (0.61–2.97)	7.92 (0.95–66.0)
40–59.9 (men), 20–39.9 (women)		2.23 (0.95–5.22)	1.08 (0.49–2.37)	4.28 (1.27–14.5)	7.98 (0.84–76.2)
≥ 60 g/day (men), ≥40 g/day (women)		1.35 (0.36–5.08)	0.87 (0.40–1.89)	–	3.32 (0.32–34.6)
Chronic disease involving pain					
Yes (ref: No)		2.69 (1.81–4.00)	3.49 (2.33–5.21)	3.23 (1.72–6.06)	4.28 (2.10–8.71)

As propionate derivatives were the more frequently consumed NSAID group, its related factors (Table 3) resembled those of non-aspirin NSAIDs: the higher the age, the lower the propionate derivative consumption, and for men, the higher the education level, the higher their propionate derivative consumption. People with conditions with chronic pain (arthritis, chronic cephalalgia or gout) used propionate derivates twice or three times more frequently than people without such conditions. Multivariate results in aspirin consumptions are displayed in Table 4. Men increased their aspirin consumption with age, being about three times higher in men older than 65, and smoking habit, with current or former smokers having 60% higher aspirin consumption than non-smoking men. In women, however, only age and number of births (OR = 1.24 for each birth) increased aspirin consumption.

Factors associated with acetate derivates are analyzed in Table 5. Chronic diseases involving pain multiplied by 2.7 (women) and 3.5 (men) the odds of having used acetate derivates. Apart from this factor, in men, only the educational level reached a positive significant relationship with consumption; men with secondary or university education reported about twice the acetate derivative consumption than men with lower educational levels. No other factor can be identified as associated with acetate derivative consumption in women. BMI displayed a non-significant association in both men and women.

### Factors related to current consumption

Non-aspirin NSAIDs consumption (Table 2) stepped down with age in both women (OR = 1.17, 1, 0.62, 0.39 and 0.46 for the ordered age groups) and men (OR = 1.57, 1, 1.27, 0.76 and 0.37). Men with university level education consumed twice as much as men with less-than-primary level; a similar result was not found in women. Consumption of non-aspirin NSAIDs was halved in current smoking men. Regarding alcohol use, only participants in the highest category of current consumption (i.e.: ≥60 g/day for men and ≥ 40 g/day for women) reported higher non-aspirin NSAID used, although estimates were unstable for women due to the small number of women in this category. Suffering arthritis, gout or chronic cephalalgia multiplies the probability of using non-aspirin NSAIDs by about 4.

Propionates consumption (Table 3) in men went down with age -reaching OR = 0.19 in men older than 75- and in current smokers (OR = 0.55). Suffering chronic pain conditions (OR = 2.48) and having reached university level (OR = 2.00) were the only factors associated with higher propionate used. In women, higher ages were also associated with lower propionate consumption (OR = 0.38 in women > 75 years) and the presence of chronic pain diseases, with higher consumption (OR = 2.58); no other factor could be identified as associated with this NSAID group.

Higher age was a risk factor for using aspirin in both women and men (Table 4), with OR for the elder group reaching 6.8 (women) and 4.7 (men). The education level was negatively associated with aspirin consumption in women, but not in men, while smokers (both former and current) were related with higher aspirin use in men. Suffering from a chronic pain condition was not associated with aspirin consumption.

Apart from chronic pain diseases, which were associated with 3 or 4 times higher acetate derivate consumption (Table 5), only non-significant positive associations were found between this NSAID group and alcohol consumption (both sexes) and higher education level (men, but not women).

### NSAID consumption and cardiovascular disease or risk factors

To further explore the risk profile of cardiovascular adverse events while taking NSAIDs, we analysed the relationship between NSAID use and current cardiovascular disease or risk factors. Results are shown in Table 6. Men with diabetes mellitus consumed less non-aspirin NSAIDs -and, specifically, less propionates- than men without that condition; no such result could be found for women. Having hypertension, hypercholesterolemia or medical history of acute coronary syndrome (i.e.: acute myocardial infarction or angina), stroke or other circulatory diseases was not associated with lower non-aspirin NSAID consumption in any gender. Using aspirin, however, increased in both women and men suffering any of these cardiovascular risk factors or diseases, with OR as higher as 15.2 (women with acute coronary syndrome), 13.3 (women having had a stroke) or 11.1 (men with acute coronary syndrome).

**Table 6:** Association between cardiovascular disease or risk factors and current NSAID consumption (odds ratios and 95% confidence intervals adjusted by age, BMI, educational level, smoking, province of recruitment and presence of arthritis, gout or chronic cephalalgia)

NSAID	Cardiovascular disease or risk factor	Women	Men
Non-aspirin NSAID	Diabetes mellitus	1.06 (0.70–1.62)	0.59 (0.37–0.95)
	Hypertension	1.02 (0.76–1.38)	0.81 (0.57–1.31)
	Hypercholesterolemia	1.13 (0.86–1.47)	1.05 (0.75–1.47)
	Acute coronary syndrome	0.64 (0.29–1.43)	0.86 (0.51–1.44)
	Stroke	1.00 (0.58–1.13)	1.11 (0.51–2.43)
	Other circulatory diseases	0.81 (0.58–1.13)	0.99 (0.63–1.58)
Propionic derivates	Diabetes mellitus	1.28 (0.79–2.06)	0.56 (0.30–1.04)
	Hypertension	0.94 (0.66–1.32)	0.81 (0.54–1.23)
	Hypercholesterolemia	1.23 (0.90–1.66)	1.06 (0.70–1.59)
	Acute coronary syndrome	1.07 (0.46–2.52)	0.63 (0.31–1.30)
	Stroke	0.85 (0.32–2.22)	0.45 (0.11–1.90)
	Other circulatory diseases	0.72 (0.49–1.08)	0.71 (0.38–1.35)
Aspirin	Diabetes mellitus	4.36 (2.39–7.94)	2.44 (1.73–3.43)
	Hypertension	4.98 (2.48–9.98)	2.44 (1.73–3.43)
	Hypercholesterolemia	2.15 (1.22–3.79)	3.62 (2.58–5.07)
	Acute coronary syndrome	15.2 (7.43–31.2)	11.1 (7.81–15.9)
	Stroke	13.3 (6.24–28.3)	2.09 (1.19–3.66)
	Other circulatory diseases	3.86 (2.16–6.90)	3.56 (2.51–5.04)
Acetic acid derivates	Diabetes mellitus	0.81 (0.33–1.99)	0.95 (0.45–2.01)
	Hypertension	1.39 (0.76–2.52)	1.01 (0.54–1.89)
	Hypercholesterolemia	1.36 (0.78–2.35)	0.84 (0.45–1.58)
	Acute coronary syndrome	–	0.69 (0.26–1.84)
	Stroke	1.77 (0.51–6.12)	1.75 (0.58–5.31)
	Other circulatory diseases	1.53 (0.83–2.80)	0.97 (0.43–2.19)

## Discussion

According to our results, NSAID consumption differed by gender and age, being aspirin more used by men and older participants and propionates by women and the youngsters. Consumption of any major NSAID group was consistently associated with educational level in men: the higher the educational level, the higher the NSAID usage. Regarding health-related behaviours, current smoker women had ever consumed more non-aspirin NSAIDs but current smoker men had lower current consumption of non-aspirin NSAIDs than no smoker women and men, respectively. Higher current consumption was found in current heavy ethanol drinkers. People with higher risk of cardiovascular adverse episodes when taking non-aspirin NSAIDs (i.e.: participants with cardiovascular disease history or cardiovascular risk factors) consumed as much non-aspirin NSAID as people without such a high risk, the only exception being men with diabetes mellitus, who halved the non-aspirin NSAID current consumption. Nonetheless, participants with higher cardiovascular risk took aspirin more frequently.

### Consumption compared with other countries

Ibuprofen was by far the more consumed NSAID in our study. Higher use of ibuprofen has also been informed in Germany [17], the US [18] and Denmark [19]. A study on 15 countries, however, reported diclofenac as the most frequently used, followed by ibuprofen [20]. Many articles have reported NSAID trend use [17, 21], which has not been analysed in our study. However, the trend in Spain seemed to be rising until 2009 and slowly decreasing from then on; specifically, ibuprofen reaching its zenith in 2009, while naproxen began to increase in 2012 [22]. Qato et al. [7] informed of increases in NSAID drugs from 2005 to 2011 in the US, with aspirin use increasing from 30.2 to 40.2% and other NSAIDs from 10.15 to 13.7%. Differences in definitions and methodology among the studies, however, makes it difficult to compare figures from different countries.

### Patterns by age and sex

Few studies reported NSAID pattern consumption by age. Like ours, Dale et al. [23] informed of an increase in aspirin and a decrease in other NSAIDs with age in Norway; Sarganas et al. [17] also reported that NSAID consumption was lower with higher age in Germany. Clinical guidelines recommend restricting non-aspirin NSAIDs in the older group because people at a higher age have higher risks of NSAID-related adverse episodes, both gastrointestinal haemorrhage and cardiovascular events [1, 3], so our results are in accordance with this. The interpretation of the age-pattern of aspirin is challenging as its consumption would be as an analgesic/anti-inflammatory drug or as anti-aggregant. In our study, aspirin was strongly associated with cardiovascular risk factors, such as hypertension and hypercholesterolemia, but especially with previous cardiovascular diseases, such as acute coronary syndrome and strokes. Aspirin is well-known as a drug able to produce gastrointestinal haemorrhage, especially in aged people, but also for its cardiovascular protective effects when used in low doses. Being in no doubt of its usage for secondary prevention in people already affected by ischemic cardiovascular disease, current US Preventive Service Task Force [24] recommendation for cardiovascular disease primary prevention, however, only supports using aspirin in people aged between 50 and 59, with possible extension on individual basis until 69 years old, but no longer as evidence of the risk/benefit relationship in patients older than 70 was considered insufficient [25]. Women consumed more NSAIDs than men, as previously reported in several articles [17, 23]. In our study, arthritis and chronic cephalalgia were more frequent in women, confirming other studies which suggested that non-malignant chronic diseases causing pain are more prevalent in women [26], eventually leading to more analgesic / anti-inflammatory usage.

### Education level

Education level could be used as a surrogate for socio-economic level. It was positively associated with NSAID use in our study. These results, however, are in contrary to those found in Germany [7, 17] which studied education and house income as different variables with similar findings: a positive association with NSAIDs: the higher the education level or the higher the house income, the more frequent the NSAID consumption is.

### Health-related behaviours

Among currently smokers, we found that women had higher ever non-aspirin NSAIDs consumption and men had lower current non-aspirin NSAIDs current or former smoker men had consumed more aspirin. Current alcohol consumption had a positive but non-significant association with non-aspirin NSAIDs. Dale et al. found a positive association of NSAID consumption with current smoking and a negative one with alcohol [23], while both alcohol and tobacco use were positively associated with NSAIDs in Sweden about 20 years previously [27]. As suggested in Dale et al., these differences may echo cultural and social changes throughout that period [23].

### Differences in current and ever NSAID consumption

Most patterns of consumption were quite similar for current and ever consumers. Non-aspirin NSAIDs were more consumed by more educated men and women, while aspirin were more consumed by older people; these patterns were more marked in current than in ever consumers, which is probably indicating recent trends. To interpret differences among current and ever consumption, however, is speculative as data on both consumptions are prone to different biases; in this regard, we focused more on similarities than on differences as similar patterns could be considered some kind of confirmatory results.

### Public health implications

Recommendations for prescribing NSAIDs have been developed in guidelines [3, 4] regarding their risk profile on gastrointestinal haemorrhage and cardiovascular episodes. They agree in considering naproxen as being less prone to cardiovascular episodes than ibuprofen, thus they recommended using naproxen if a non-aspirin NSAID is needed in patients with high cardiovascular risk, especially if they are taking aspirin for cardio-protection. However, the FDA (Food and Drug Administration), in a safety announcement for advising on cardiovascular risks associated with NSAIDs, stated there is not enough evidence to determine that a specific non-aspirin NSAID has higher or lower cardiovascular risk than any other [5]. Therefore, the relevance of higher use of ibuprofen than naproxen -as we reported- is unclear. On the other hand, older people, which are at high risk of cardiovascular events or gastrointestinal haemorrhage, tend to use less non-aspirin NSAIDs and more aspirin than people at low risk. Finally, although the higher consumption of aspirin by people with cardiovascular diseases or risk factors would be related with its usage as secondary prevention, the fact that the same high cardiovascular risk people did not report lower non-aspirin NSAID consumption brings up a point of concern because these NSAIDs could put them at higher cardiovascular risk.

### Strenghs and limitations

Our study has some limitations. Firstly, information on NSAID use was obtained in a face-to-face interview, so it would be affected by recall bias and social desirability bias as well; moreover, no information was recorded on prescriptions in order to validate whether the information provided in the interview accurately represented actual NSAID consumption. On the other hand, some subjects could consider that drugs obtained over-the-counter -as occurs with many NSAID, especially aspirin- are not really medicines, leading to underreport their usage. Secondly, the sample is formed by the control group in a case-control study on several types of cancer; although participants in the study were selected at random, some cancer cases had to be excluded. This fact could bias the results towards lower NSAID consumption than the general population. Thirdly, although our subjects were selected at random, we cannot exclude that people agreeing to participate could be self-selected because of their health behaviours or interests, which could limit the generalization of our results. Fourth, our data did not allow us to distinguish whether aspirin is being taken as painkiller or for cardiovascular prevention purposes.

By the other hand, our study has also some strengths within his study. Firstly, we have a large sample from 12 different Spanish provinces, which makes our results more reliable. Secondly, the vast amount of information gathered as part of a case-control study on cancer allows us the analysis of determinants of NSAID consumption. It is noteworthy that participants were not aware of any hypothesis regarding NSAID usage when they were interviewed, which makes unlikely the presence of differential biases associated with the reported informations.

## Conclusion

Summarizing, we found that propionates are the most consumed group of NSAIDs in Spain. Consumption of non-aspirin NSAIDs was associated with demographic groups with lower gastrointestinal and cardiovascular risk; however, participants at high cardiovascular risk had no lower non-aspirin NSAID consumption, which points out some concerns on the current NSAID consumption or prescription in Spain.

## Additional files

Additional file 1: NSAID-group ever use by age and sex [%, (95% CI)]. (PDF 47 kb)

Additional file 2: NSAID-group current use by age and sex [%, (95% CI)]. (PDF 43 kb)

Additional file 3: NSAID ever group use by province of recruitment [%, (95% CI)]. (PDF 21 kb)

Additional file 4: Consumption of specific non-aspirin NSAIDs. Only active principles with reported anytime-consumption over 1% are included. Data indicate percentage and 95% confidence interval. (PDF 36 kb)

## Abbreviations

ATC: Therapeutic Classification of Drugs
CEIC: Comité Ético de Investigación Clínica
CI: % confidence intervals
FDA: Food and Drug Administration
FFQ: Food Frequency Questionnaire
GI: Gastrointestinal
MCC: Multicase-control
NSAIDs: Non-steroidal anti-inflammatory drugs
OR: Odds ratios
