# Supplementary material for: Epidemiology of non-steroidal anti-inflammatory drugs consumption in Spain. The MCC-Spain study
Source: BMC Public Health. 2018 Sep 21;18:1134. doi: 10.1186/s12889-018-6019-z (PMC6150967; doi:10.1186/s12889-018-6019-z)
Supplement: Supplementary file 1 — NSAID-group ever use by age and sex [%, (95% CI)]. (PDF 47 kb) [file 12889_2018_6019_MOESM1_ESM.pdf]

Additional file 1. NSAID-group ever use by age and sex [%, (95% CI)]

		**Non-aspirin NSAIDs (M01a)**	**Aspirin (N02ba01)**	**Butylpyrazolidines (M01aa)**	**Acetate derivates (M01ab)**	**Oxicam (M01ac)**	**Propionates (M01ae)**	**Coxib (M01ah)**	**Others (M01ax)**
**All ages**	**ALL**	29.6 (28.2-31.1)	11.2 (10.2-12.2)	0.03 (0.00-0.15)	7.6 (6.8-8.5)	0.6 (0.4-0.9)	20.9 (19.6-22.3)	0.6 (0.4-1.0)	2.2 (1.8-2.7)
	**Men**	22.3 (20.5-24.2)	11.7 (10.3-13.2)	0.1 (0.0-0.4)	7.3 (6.2-8.5)	0.4 (0.2-0.8)	14.8 (13.3-16.4)	0.3 (0.1-0.7)	1.7 (1.2-2.4)
	**Women**	38.8 (36.7-41.0)	5.2 (4.3-6.3)	0	8.5 (7.3-9.8)	0.8 (0.5-1.3)	29.2 (27.3-31.3)	1.0 (0.6-1.5)	2.5 (1.8-3.2)
**<45**	**All**	44.2 (39.4-49.1)	1.7 (0.7-3.4)	0	4.8 (2.9-7.2)	0.5 (0.1-1.7)	39.7 (35.0-44.5)	0.2 (0.0-1.3)	0.7 (0.1-2.1)
	**Men**	37.5 (25.7-50.5)	1.6 (0.1-8.4)	0	6.3 (1.7-15.2)	0	34.4 (22.9-47.3)	0	0
	**Women**	45.4 (40.1-50.7)	1.7 (0.6-3.6)	0	4.5 (2.6-7.2)	0.6 (0.1-2.0)	40.6 (35.5-45.9)	0.3 (0.0-1.6)	0.8 (0.2-2.4)
**45-54**	**All**	40.8 (36.7-44.9)	3.1 (1.9-4.9)	0	8.1 (6.0-10.6)	0.7 (0.2-1.8)	33.5 (29.7-37.5)	0.9 (0.3-2.0)	1.7 (0.8-3.2)
	**Men**	33.8 (26.3-41.9)	5.3 (2.3-10.2)	0	9.9 (5.7-15.9)	0.7 (0.0-3.6)	27.2 (20.2-35.0)	0	0.7 (0.0-3.6)
	**Women**	43.2 (38.4-48.1)	2.3 (1.1-4.3)	0	7.5 (5.2-10.4)	0.7 (0.1-2.0)	35.7 (31.2-40.5)	1.2 (0.4-2.7)	2.1 (1.0-4.0)
**55-64**	**All**	30.1 (27.3-33.0)	7.6 (6.0-9.4)	0	8.9 (7.2-10.8)	0.6 (0.2-1.3)	20.9 (18.5-23.6)	1.0 (0.5-1.8)	2.4 (1.5-3.5)
	**Men**	23.6 (20.1-27.3)	9.1 (6.9-11.8)	0	7.7 (5.7-10.2)	0.5 (0.1-1.5)	15.3 (12.4-18.5)	0.7 (0.2-1.8)	2.1 (1.1-3.7)
	**Women**	38.5 (34.0-43.2)	5.6 (3.7-8.2)	0	10.4 (7.7-13.6)	0.7 (0.1-2.0)	28.2 (24.0-32.6)	1.4 (0.5-2.9)	2.7 (1.4-4.7)
**65-74**	**All**	27.4 (24.9-30.0)	11.4 (9.7-13.3)	0.2 (0.0-0.6)	8.2 (6.8-9.9)	0.6 (0.3-1.2)	17.7 (15.6-19.9)	0.5 (0.2-1.1)	2.7 (1.9-3.8)
	**Men**	22.7 (19.8-25.7)	14.1 (11.8-16.7)	0	7.5 (5.9-9.5)	0.4 (0.1-1.1)	14.6 (12.2-17.2)	0.4 (0.1-1.1)	1.9 (1.0-3.1)

	**Women**	35.3 (31.0-39.8)	6.9 (4.8-9.6)	0	9.5 (7.0-12.4)	1.1 (0.3-2.4)	22.9 (19.2-26.9)	0.8 (0.2-2.1)	4.2 (2.6-6.4)
**≥75**	**All**	21.1 (18.3-24.2)	12.4 (10.1-14.9)	0	7.5 (5.7-9.6)	0.5 (0.1-1.3)	12.3 (10.1-14.9)	0.5 (0.1-1.3)	1.7 (0.9-2.9)
	**Men**	14.2 (11.1-17.7)	14.2 (11.1-17.7)	0	5.5 (3.6-8.1)	0.2 (0.0-1.2)	7.7 (5.5-10.6)	0	1.5 (0.6-3.2)
	**Women**	31.1 (26.1-36.5)	9.8 (6.7-13.6)	0	10.4 (7.3-14.3)	0.9 (0.2-2.7)	18.9 (14.7-23.6)	1.3 (0.3-3.2)	1.9 (0.7-4.1)
