# Supplementary material for: Epidemiology of non-steroidal anti-inflammatory drugs consumption in Spain. The MCC-Spain study
Source: BMC Public Health. 2018 Sep 21;18:1134. doi: 10.1186/s12889-018-6019-z (PMC6150967; doi:10.1186/s12889-018-6019-z)
Supplement: Supplementary file 2 — NSAID-group current use by age and sex [%, (95% CI)]. (PDF 43 kb) [file 12889_2018_6019_MOESM2_ESM.pdf]

Additional file 2. NSAID-group current use by age and sex [%, (95% CI)]

		**Non-aspirin NSAIDs (M01a)**	**Aspirin (N02ba01)**	**Butylpyrazolidines (M01aa)**	**Acetate derivates (M01ab)**	**Oxicam (M01ac)**	**Propionates (M01ae)**	**Coxib (M01ah)**	**Others (M01ax)**
**All ages**	**ALL**	14.8 (13.7-15.9)	5.9 (5.2-6.7)	0	2.7 (2.2-3.3)	0.2 (0.1-0.5)	10.3 (9.4-11.3)	0.5 (0.3-7.3)	1.6 (1.2-2.0)
	**Men**	9.0 (7.8-10.3)	9.0 (7.8-10.3)	0	2.3 (1.7-3.1)	0.1 (0.0-0.4)	5.6 (4.7-6.7)	0.3 (0.1-0.6)	1.3 (0.9-1.9)
	**Women**	20.7 (19.0-22.5)	2.9 (2.2-3.7)	0	3.1 (2.4-4.0)	0.3 (0.1-0.7)	15.0 (13.4-16.6)	0.6 (0.3-1.1)	1.9 (1.3-2.6)
**<45**	**All**	22.1 (18.2-26.4)	0.2 (0.0-1.3)	0	1.7 (0.7-3.4)	0.2 (0.0-1.3)	19.0 (15.4-23.1)	0.2 (0.0-1.3)	0.7 (0.1-2.1)
	**Men**	10.9 (4.5-21.2)	0.0 (0.0-0.6)	0	3.1 (0.4-10.8)	0	9.4 (3.5-19.3)	0	0
	**Women**	24.1 (19.7-28.9)	0.3 (0.0-1.6)	0	1.4 (0.5-3.2)	0.3 (0.0-1.6)	20.7 (16.6-25.3)	0.3 (0.0-1.6)	0.8 (0.2-2.4)
**45-54**	**All**	21.4 (18.1-25.0)	1.0 (0.4-2.2)	0	2.6 (1.5-4.2)	0.2 (0.0-1.0)	17.1 (14.1-20.4)	0.3 (0.0-1.2)	1.4 (0.6-2.7)
	**Men**	9.3 (5.2-15.1)	2.6 (0.7-6.6)	0	2.0 (0.4-5.7)	0	7.9 (4.2-13.5)	0	0.7 (0.0-3.6)

	**Women**	25.7 (21.6-30.1)	0.5 (0.1-1.7)	0	2.8 (1.5-4.8)	0.2 (0.0-1.3)	20.3 (16.6-24.5)	0.5 (0.1-1.7)	1.6 (0.7-3.3)
**55-64**	**All**	15.0 (12.9-17.4)	5.2 (3.9-6.8)	0	2.9 (1.9-4.1)	0.3 (0.1-0.9)	10.5 (8.6-12.5)	0.7 (0.3-1.4)	1.7 (1.0-2.7)
	**Men**	11.6 (9.1-14.5)	7.0 (5.1-9.4)	0	2.6 (1.5-4.3)	0.2 (0.0-1.0)	7.2 (5.2-9.6)	0.5 (0.1-1.5)	1.8 (0.8-3.2)
	**Women**	19.4 (15.8-23.4)	2.9 (1.6-5.0)	0	3.2 (1.7-5.2)	0.5 (0.1-1.6)	14.6 (11.5-18.3)	0.9 (0.2-2.3)	1.6 (0.6-3.2)
**65-74**	**All**	12.0 (10.2-13.9)	8.5 (7.0-10.2)	0	2.7 (1.9-3.8)	0.2 80.0-0.7)	7.1 (5.8-8.7)	0.4 (0.1-0.9)	2.1 (1.4-3.1)
	**Men**	9.1 (7.2-11.3)	11.0 (8.9-13.3)	0	2.2 (1.3-3.5)	0.2 (0.0-0.9)	5.5 (4.0-7.3)	0.4 (0.1-1.1)	1.4 (0.7-2.4)
	**Women**	16.8 (13.6-20.5)	4.4 (2.8-6.7)	0	3.6 (2.1-5.7)	0.2 (0.0-1.2)	9.9 (7.3-12.9)	0.4 (0.1-1.5)	3.4 (1.9-5.4)
**≥75**	**All**	10.4 (8.3-12.8)	9.4 (7.4-11.6)	0	3.1 (2.1-4.6)	0.3 (0.0-0.9)	5.5 (4.0-7.3)	0.5 (0.1-1.3)	1.3 (0.6-2.4)
	**Men**	5.1 (3.3-7.5)	11.3 (8.5-14.6)	0	2.0 (0.9-3.7)	0	2.7 (1.4-4.6)	0	1.1 (0.4-2.6)
	**Women**	18.9 (13.9-22.6)	6.6 (4.1-9.9)	0	4.7 (2.7-7.7)	0.6 (0.1-2.3)	9.4 (6.5-13.2)	1.3 (0.3-3.2)	1.6 (0.5-3.6)
