# Supplementary material for: Epidemiology of non-steroidal anti-inflammatory drugs consumption in Spain. The MCC-Spain study
Source: BMC Public Health. 2018 Sep 21;18:1134. doi: 10.1186/s12889-018-6019-z (PMC6150967; doi:10.1186/s12889-018-6019-z)
Supplement: Supplementary file 3 — NSAID ever group use by province of recruitment [%, (95% CI)]. (PDF 21 kb) [file 12889_2018_6019_MOESM3_ESM.pdf]

Additional file 3. NSAID ever group use by province of recruitment [%, (95% CI)]

PROVINCE	Non-aspirin NSAIDs (M01a)	Aspirin (N02ba01)	Butylpyrazolidines (M01aa)	Acetate derivates (M01ab)	Oxicam (M01ac)	Propionates (M01ae)	Coxib (M01ah)	Others (M01ax)
**Asturias**	32.3 (26.4-38.8)	6.9 (4.0-11.0)	0	4.7 (2.4-8.3)	0	22.8 (17.6-28.8)	1.3 (0.3-3.7)	2.6 (1.0-5.5)
**Barcelona**	43.1 (40.0-46.2)	11.7 (9.8-13.9)	0.2 (0.0-0.7)	15.8 (13.6-18.2)	0.5 (0.2-1.2)	33.1 (30.2-36.1)	0.5 (0.2-1.2)	3.2 (2.2-4.5)
**Cantabria**	26.7 (22.3-31.5)	6.3 (4.1-9.3)	0	3.7 (2.0-6.1)	1.1 (0.3-2.7)	18.8 (15.0-23.1)	1.3 (0.4-3.1)	1.9 (0.7-3.8)
**Girona**	45.1 (34.1-56.5)	6.1 (2.0-13.7)	0	8.5 (3.5-16.8)	1.2 (0.0-6.6)	37.8 (27.3-49.2)	2.4 (0.3-8.5)	1.2 (0.0-6.6)
**Granada**	13.4 (8.8-19.1)	2.7 (0.9-6.1)	0	2.7 (0.9-6.1)	0	11.2 (7.1-16.7)	0	1.1 (0.1-3.8)
**Gipuzkoa**	23.5 (19.2-28.2)	3.3 (1.7-5.7)	0	7.7 (5.2-11.0)	0.8 (0.2-2.4)	13.3 (9.9-17.2)	1.1 (0.3-2.8)	2.5 (1.1-4.7)

**Huelva**	33.1 (26.3-40.6)	14.0 (9.3-20.0)	0	9.0 (5.2-14.2)	0.6 (0.0-3.1)	27.0 (20.6-34.1)	0.6 (0.0-3.1)	0.6 (0.0-3.1)
**León**	31.7 (27.4-36.3)	6.8 (4.6-9.6)	0	4.3 (2.6-6.6)	0.2 (0.0-1.3)	23.4 (19.5-27.6)	0.2 (0.0-1.3)	1.6 (0.6-3.2)
**Madrid**	22.0 (19.0-25.1)	8.7 (6.8-11.0)	0	2.9 (1.8-4.3)	0.5 (0.01-1.4)	14.9 (12.4-17.7)	0	1.9 (1.0-3.2)
**Murcia**	16.7 (7.0-31.4)	9.5 (2.7-22.6)	0	7.1 (1.5-19.5)	2.4 (0.1-12.6)	9.5 (2.7-22.6)	0	0
**Navarra**	29.6 (24.2-35.3)	10.6 (7.2-14.8)	0	12.8 (9.1-17.3)	1.5 (0.4-3.7)	16.4 (12.2-21.4)	1.5 (0.4-3.7)	0.4 (0.01-2.02)
**Valencia**	25.2 (18.5-32.8)	7.7 (4.1-13.1)	0	1.9 (0.4-5.6)	0	3.2 (1.4-27.2)	0.9 (0.2-4.6)	3.2 (1.1-7.4)
