# Supplementary material for: Epidemiology of non-steroidal anti-inflammatory drugs consumption in Spain. The MCC-Spain study
Source: BMC Public Health. 2018 Sep 21;18:1134. doi: 10.1186/s12889-018-6019-z (PMC6150967; doi:10.1186/s12889-018-6019-z)
Supplement: Supplementary file 4 — Consumption of specific non-aspirin NSAIDs. Only active principles with reported anytime-consumption over 1% are included. Data indicate percentage and 95% confidence interval. (PDF 36 kb) [file 12889_2018_6019_MOESM4_ESM.pdf]

Additional file 4. Consumption of specific non-aspirin NSAIDs. Only active principles with reported anytime-consumption over 1% are included. Data indicate percentage and 95% confidence interval

**NSAID group**	**NSAID specific drug**	**Anytime consumption**	**Current consumption**
**Acetate derivates**	**Diclofenac**	6.4 (5.6-7.2)	2.0 (1.6-2.4)
	**Aciclofenac**	1.5 (1.1-1.9)	0.6 (0.4-0.9)
**Propionates**	**Ibuprofen**	20.4 (19.2-21.7)	9.2 (8.4-10.2)
	**Naproxen**	1.4 (1.0-1.8)	0.7 (0.5-1.0)
**Others**	**Chondroitin sulphate**	1.3 (0.9-1.6)	1.0 (0.7-1.3)
